# Efficacy and safety of a mineral and vitamin treatment on symptoms of antenatal depression: 12-week fully blinded randomised placebo-controlled trial (NUTRIMUM)

**DOI:** 10.1192/bjo.2024.706

**Published:** 2024-06-03

**Authors:** Hayley A. Bradley, Elena Moltchanova, Roger T. Mulder, Lesley Dixon, Jacki Henderson, Julia J. Rucklidge

**Affiliations:** School of Psychology, Speech and Hearing, University of Canterbury, New Zealand; School of Mathematics and Statistics, University of Canterbury, New Zealand; Department of Psychological Medicine, University of Otago, New Zealand; New Zealand College of Midwives, New Zealand

**Keywords:** Perinatal psychiatry, vitamin, mineral, micronutrients, antenatal depression

## Abstract

**Background:**

Broad-spectrum micronutrients (minerals and vitamins) have shown benefit for treatment of depressive symptoms.

**Aims:**

To determine whether additional micronutrients reduce symptoms of antenatal depression.

**Method:**

Eighty-eight medication-free pregnant women at 12–24 weeks gestation, who scored ≥13 on the Edinburgh Postnatal Depression Scale (EPDS), were randomised 1:1 to micronutrients or active placebo (containing iodine and riboflavin), for 12 weeks. Micronutrient doses were generally between recommended dietary allowance and tolerable upper level. Primary outcomes (EPDS and Clinical Global Impression – Improvement Scale (CGI-I)) were analysed with constrained longitudinal data analysis.

**Results:**

Seventeen (19%) women dropped out, with no group differences, and four (4.5%) gave birth before trial completion. Both groups improved on the EPDS, with no group differences (*P* = 0.1018); 77.3% taking micronutrients and 72.7% taking placebos were considered recovered. However, the micronutrient group demonstrated significantly greater improvement, based on CGI-I clinician ratings, over time (*P* = 0.0196). The micronutrient group had significantly greater improvement on sleep and global assessment of functioning, and were more likely to identify themselves as ‘much’ to ‘very much’ improved (68.8%) compared with placebo (38.5%) (odds ratio 3.52, *P* = 0.011; number needed to treat: 3). There were no significant group differences on treatment-emergent adverse events, including suicidal ideation. Homocysteine decreased significantly more in the micronutrient group. Presence of personality difficulties, history of psychiatric medication use and higher social support tended to increase micronutrient response compared with placebo.

**Conclusions:**

This study highlights the benefits of active monitoring on antenatal depression, with added efficacy for overall functioning when taking micronutrients, with no evidence of harm. Trial replication with larger samples and clinically diagnosed depression are needed.

Antenatal depression affects 15–21% of pregnant women worldwide,^1^ and increases the risk of pregnancy, birth and neonatal complications, as well as postnatal depression.^2–4^ It has also been associated with emotional, behavioural and development problems in the offspring.^5^ In most countries, psychological treatments, such as cognitive–behavioural therapy and interpersonal psychotherapy, are recommended for pregnant women experiencing depression.^6^ However, women often do not access these treatments because of issues with time, cost, stigma and childcare.^7^ For those with more severe symptoms or where psychological interventions have not been effective or are not appropriate, antidepressant medication is recommended.^6^ Risks associated with antidepressant exposure *in utero* include preterm delivery, higher admission rates to specialised care and low birth weight,^8^ and pregnant women are often reluctant to take them.^9^ The impact of antenatal depression and the limitations of treatment options highlight the need for more accessible and safe interventions.

## Nutrition and mental health

There is increased interest in the role of nutrition in alleviating depression, with international guidelines recommending lifestyle changes, which includes a healthy diet, as foundational in the treatment of mood disorders.^6^ Other research highlights the potential benefit of additional micronutrients (vitamins and minerals) in pill form to improve depression, with a good safety profile.^10–13^ The rationale for providing additional micronutrients includes poverty of diet, inborn errors of metabolism, supporting mitochondrial activity and methylation, and reducing oxidative stress, gut dysbiosis and inflammation.^11^ Although there has been one randomised controlled trial (RCT)^14^ on micronutrients during pregnancy and their beneficial effects on mood and quality of life, the study did not directly recruit for depression and only recruited women with HIV. Additionally, analyses on mental health outcomes were secondary to the original study aim, and there were no minerals contained within the intervention. Other RCTs with micronutrients during pregnancy focus on pre-conception supplementation, those with subclinical symptoms, postpartum functioning or infant outcomes.

The current study, NUTRitional Intervention for Maternal difficUlties in Mental health (NUTRIMUM), is the first RCT specifically designed to assess the efficacy and safety of a broad-spectrum micronutrient formula, given in doses generally between the recommended dietary allowance and tolerable upper intake level, on symptoms of antenatal depression and global functioning in a pregnancy cohort. The micronutrient formula used has demonstrated benefit for a variety of mental health concerns in non-pregnant adults and children, in addition to showing good safety and tolerability.^11^

## Method

### Study design

NUTRIMUM was a 12-week, fully blinded trial in which participants were randomised 1:1 to micronutrients or active placebo. The authors assert that all procedures contributing to this work comply with the ethical standards of the relevant national and institutional committees on human experimentation and with the Helsinki Declaration of 1975, as revised in 2008. This human study was approved by Southern Human and Disabilities Ethics Committee and University of Canterbury Human Ethics Committee (joint approval: 16/STH/187). The clinical trial was also approved by the Standing Committee on Therapeutic Trials (approval number: 16/SCOTT/131). Participant registration took place from 12 April 2017 to 5 October 2020. All participants provided written informed consent to participate in this study. See Bradley et al^15^ for a detailed methodology. The trial was prospectively registered at www.anzctr.org.au (identifier: ACTRN12617000354381).

### Participants

**Fig. 1:**
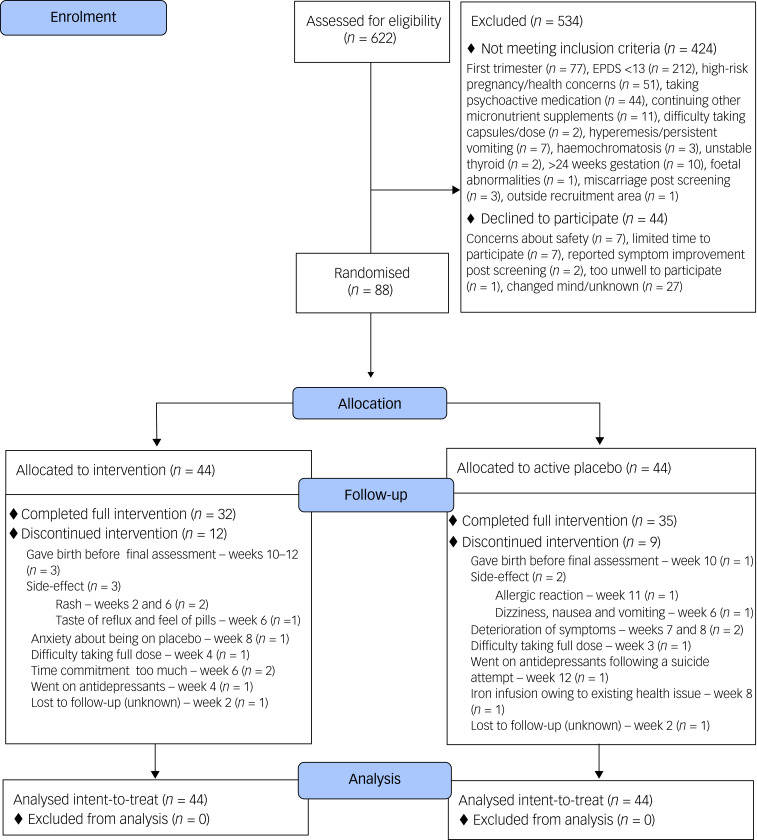
Consolidated Standards of Reporting Trials diagram displaying the flow of participants through the trial. EPDS, Edinburgh Postnatal Depression Scale.

Women were initially recruited from the Canterbury region of New Zealand, via social media, advertisements in radiology clinics and direct referrals from midwives. After the first COVID-19 lockdown in March 2020, face-to-face assessments were conducted via telephone/video call, enabling national recruitment. Nine participants were recruited from outside of Canterbury. Pregnant women were eligible if they were deemed reliable and adherent to treatment, at 12–24 weeks gestation, aged ≥16 years, had a low-risk singleton pregnancy, had not taken psychiatric medication for 4 weeks and scored ≥13 on the Edinburgh Postnatal Depression Scale (EPDS) (Fig. 1). Exclusion criteria included regular vomiting, current/recent significant pregnancy complications, known foetal abnormalities, serious current or historical medical conditions, known allergy to intervention ingredients, known metabolic condition, known neurological disorder, untreated or unstable thyroid disease, and/or desire to continue taking antenatal supplements not required for medical purposes (decisions discussed and made on a case-by-case basis). Some women continued or started single nutrients (e.g. iron) if medically indicated.

Participants could continue any form of psychotherapy that had begun before screening (*n* = 12, seven in the micronutrient group and five in the active placebo group). Also, they continued to access usual care for their pregnancy. In Aotearoa, New Zealand, once pregnant, the person will normally register during the first trimester with a midwife Lead Maternity Carer, who is responsible for care throughout the pregnancy, labour and birth, and postpartum (up to 6 weeks after birth). The pregnant person and the baby's health are assessed on a monthly basis as the pregnancy progresses.

### Randomisation and masking

A research assistant not involved in any other aspect of the study generated the random allocation sequence in permuted blocks of four, using www.randomization.com. The capsules were packaged and numbered by an independent pharmacist in white opaque bottles containing two vanilla sachets, to ensure all capsules smelled similar. Individual opaque envelopes containing each participant's randomisation group was available to the research team in case breaking the blind was required for a medical emergency. All participants, researchers and statisticians were blind to participants' treatment condition.

The formula, Daily Essential Nutrients, was provided without cost by Hardy Nutritionals, along with visually identical capsules for placebo containing 0.1 mg of riboflavin per capsule, to mimic the change in colour of urine caused by B-vitamins. Pill containers, provided to assist with adherence and recall, also contained vanilla sachets in each compartment to mask any possible difference in smell.

### Procedures

Eligible participants were invited to attend an initial appointment to discuss the details of the study either in person or via telephone/video call for those residing outside of Canterbury. Following written informed consent, a baseline appointment was booked, which involved a series of clinician-rated and self-reported assessments (see measures). Participants completed self-reported measures every 2 weeks online, and either visited or spoke with the clinician via telephone or video call once a month to complete clinician measures. A new bottle of capsules was provided each month and old bottles with unused capsules were returned (either in person or posted). Participants were given a NZD$10 petrol voucher for in-person research visits (no compensation was provided for online/telephone meetings), to reimburse travel expenses, and a gift hamper was given at birth (worth NZ$50.00). Upon trial completion, both assessors and participants were asked to guess what treatment condition they thought participants had been allocated to.

Taken at the full dose, both arms provided the daily supplemental intake of 150 mcg of iodine for pregnancy, the only nutrient supplement recommended at the time of the study by the New Zealand Ministry of Health to take after 12 weeks gestation. See Table 1 for ingredients and doses.

**Table 1:** Ingredients of micronutrient intervention and active placebo

Daily Essential Nutrients supplement facts: total dose per 12 capsules (daily dose taken as four capsules three times a day)	
Vitamin A (as retinyl palmitate)	5760 IU
Vitamin C (as ascorbic acid)	600 mg
Vitamin D (as cholecalciferol)	3000 IU
Vitamin E (as d-alpha tocopheryl succinate)	360 IU
Vitamin K (75% as phylloquinone; 25% as menaquinone-7)	120 mcg
Thiamine (as thiamine mononitrate)	60 mg
Riboflavin	18 mg
Niacin (as niacinamide)	90 mg
Vitamin B6 (as pyridoxine hydrochloride)	69.9 mg
Folate (as L-methylfolate calcium)	801 mcg
Vitamin B12 (as methylcobalamin)	900 mcg
Biotin	1080 mcg
Pantothenic acid (as d-calcium pantothenate)	30 mg
Calcium (as chelate)	1320 mg
Iron (as chelate)	13.8 mg
Phosphorus (as chelate)	840 mg
Iodine (as chelate)	204 mcg
Magnesium (as chelate)	600 mg
Zinc (as chelate)	48 mg
Selenium (as chelate)	204 mcg
Copper (as chelate)	7.2 mg
Manganese (as chelate)	9.6 mg
Chromium (as chelate)	624 mcg
Molybdenum (as chelate)	144 mcg
Potassium (as chelate)	240 mg
Proprietary blend: choline bitartrate, alpha-lipoic acid, mineral wax, inositol, acetyl-L-carnitine, grape seed extract, ginkgo biloba leaf extract, L-methionine, *N*-acetyl-L-cysteine, boron (as chelate), vanadium (as chelate), lithium orotate (as chelate), nickel (as chelate), Other ingredients: vegetarian capsule (hypromellose), microcrystalline cellulose, magnesium stearate, silicon dioxide, titanium dioxide	
Active placebo supplement facts: total dose per 12 capsules (daily dose taken as four capsules three times a day)	
Riboflavin	1.2 mg
Iodine (given as potassium iodide)	150 mcg
Silicon dioxide	30 mg
Magnesium stearate	60 mg
Microcrystalline cellulose	6 g

Participants were expected to consume 12 capsules, taken as four capsules, three times a day, with food and water, and to report missed doses. Titration to the full dose occurred over 7 days, whereby participants initially took one capsule, three times a day for 2 days. The dose increased by three capsules every second day.

### Outcomes

#### Primary outcomes

The EPDS,^16^ a ten-item self-report questionnaire, assesses symptoms of depression during pregnancy and was administered every 2 weeks online. Total scores ranged from 0 to 30, with higher scores indicating greater levels of distress. A cut-off ≥13 identifies women in the moderate range of depression and has demonstrated high specificity with major depressive disorder (0.95),^17^ suggesting the EPDS can accurately identify antenatal depression. The EPDS was originally developed for use during the postpartum period; however, it has since been validated for use in pregnancy,^18^ and is the most commonly validated tool for identifying depression during the antenatal period.^19^ It has also been widely used in studies examining treatment efficacy for antenatal depression.^20,21^

The Clinical Global Impressions – Improvement Scale (CGI-I)^22^ is a clinician-rated assessment of change in participant's overall symptoms from baseline ranging from 1 (very much improved) to 7 (very much worse). A score of 4 identifies either baseline or no change. The CGI-I was completed every 4 weeks (three times) by postgraduate clinical psychology students, under the supervision of a registered clinical psychologist, and the rating was based on all available data collected at monthly appointments.

#### Secondary outcomes

Secondary outcomes were scores on the (a) Clinician-administered Montgomery–Åsberg Depression Rating Scale (MADRS)^23^ and Global Assessment of Functioning (GAF)^24^ (both administered once a month); and (b) participant-rated Generalised Anxiety Disorder-7 (GAD-7),^25^ Perceived Stress Scale (PSS),^26^ Short-Form Health Survey (SF-12),^27^ Difficulties in Emotion Regulation Scale – Short-Form (DERS-SF),^28^ Depression, Anxiety and Stress Scale (DASS-21),^29^ and Pittsburgh Sleep Quality Index (PSQI),^30^ all administered online every 2 weeks or once a month from baseline to end of trial. Participants were also asked at the end of the trial to rate their own improvement via the CGI-I modified for participant report (M-CGI-I), a commonly used adaptation in treatment research.^31^

#### Safety and adherence

Adverse events were recorded online every 2 weeks by the participants, based on the modified Antidepressant Side-Effect Checklist (ASEC).^32^ Haematology blood count, nutrient levels, weight, blood pressure and pulse were taken at baseline and at trial completion in Canterbury-residing participants. Treatment adherence was measured by total number of capsules taken during the trial, as reported by participants and counting returned capsules. A participant was deemed as adherent if they took at least 70% of the assigned capsules.

#### Other measures

Demographic characteristics (age, ethnicity, socioeconomic status (determined by the New Zealand Socio-Economic Index (NZSEI)), household income and relationship status), perceived social support (determined by the Multidimensional Scale of Perceived Social Support (MSPSS),^33^ diet^26^ and psychiatric treatment history were collected at baseline from the participants. The Structured Clinical Interview for DSM-5 (Research Version) (SCID-5)^34^ was administered by the clinician to determine presence of current and historical mood and anxiety disorders, as well as the Standardised Assessment of Personality – Abbreviated Scale (SAPAS),^35^ a measure used to identify presence of personality difficulties (identified by a score of ≥3). Alcohol^36^ and nicotine^37^ intake was assessed based on self-report. The Helping Alliance Questionnaire (HAQ)^38^ measured the extent to which a participant developed an alliance with the clinician.

### Statistical analysis

A sample size of 45 per group was expected to detect a clinically important average difference of *d* = 0.6 at the last time point between micronutrients and placebo, with 80% power at *P* < 0.05 (two-sided), a difference observed using these same nutrients in non-pregnant populations, as no data were available to inform power within pregnancy.^15^ When allowing for 30% drop-out, the target total sample size was 120 participants; however, difficulties with recruitment and the COVID-19 pandemic led to early termination of the trial, reducing potential power to detect differences, although intent-to-treat and mixed-effects linear models mitigated potential loss of power from drop-out such that our final sample of 88 was large enough to detect medium effects.

Mixed-effects (generalised) linear models with patient-specific random effects to account for repeated measures were implemented in R, version 4.0.5 for Windows (R Core Team, Vienna, Austria; see https://www.R-project.org/.) via *lme4* and *lmer4Test* packages.^39,40^ The responses at baseline were assumed to be identical (the so-called constrained longitudinal data analysis model).^41^ Moreover, for CGI-I, based on scoring convention, the baseline was fixed at 4 for all participants. To improve the model fit and promote normality and heteroscedasticity of both residuals and random effects, the scale responses were logged. If the scale response started from 0, it was shifted by 1.

After the relevant transformation, the model for each expected response Y is formulated as:
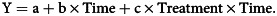


In addition to fitting the model with only time, treatment and their interaction as covariates, for each outcome, we utilised a covariate-adjusted model to explore the interaction of treatment group with time and with a number of variables informed by the literature as possible predictors of treatment response (see study protocol^15^ for more detail). These included age and gestational age,^42^ past psychiatric medication use,^43^ socioeconomic status (NZSEI score),^44,45^ personality difficulties (SAPAS ≥3)^46^ and perceived social support (MSPSS score).^47^ Some measured predictors were not included because of missing data and/or floor effects. The covariate-adjusted model for each expected response Y with covariate x is formulated as:
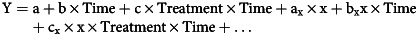
with as many additional covariate-specific terms as necessary.

The effect of treatment and of individual covariates was tested by comparing the amount of variance explained by the covariate-adjusted model above to the variance explained by the same model without the treatment or the covariate in question via a *χ*^2^-test. The number of individual person-time point observations available for each analysed variable varied from 222 to 478, except for M-CGI-I, where only 71 observations were available (Table 4).

Self-reported CGI-I (M-CGI-I) was only available at the end of the trial (*n* = 71). A log-linear regression was fitted to test the effects of treatment with and without covariates. For binarized measures (treatment responders, see below), a generalised linear model with a binary response was fitted.

Initially, it was intended to apply the last observation carried forward method to missing data. However, a growing body of literature indicates this methodology is based on unrealistic assumptions and likely to lead to biased results.^48^ The full information maximum likelihood, as suggested by Li and Stuart,^49^ was used instead. It was also intended to control for baseline in our modelling; however, further consideration led us to use constrained longitudinal data analysis (cLDA) as the better choice over analysis of covariance and longitudinal data analysis,^50,51^ i.e. to impose equality between treatment arm-specific response at baseline rather than use baseline as a covariate. Per-protocol analysis of the primary outcomes was also intended; however, this was not conducted because of the likelihood of overestimating treatment effects.

#### Clinical significance

A change of ≥4 points and a score of ≤12 at trial completion was considered a reliable and clinically significant change on the EPDS. Treatment responder was also determined based on the clinician and participant CGI-I ratings, determined by a score of 1 (very much improved) or 2 (much improved) on the CGI-I/M-CGI-I at the end of the trial.

#### Adverse events

To analyse differences in adverse events between micronutrients and active placebo, first, any symptom reported at baseline (score of ≥1 on the modified ASEC or self-reported at baseline) was removed from the analysis because it is not possible to ascertain whether these symptoms were treatment-emergent, as they were already present before treatment started. Second, any adverse event that emerged during treatment was considered present when it occurred in ≥5% of participants who did not report the symptom at baseline within each treatment group. The prevalence of adverse events was compared between groups with *χ*^2^-tests. Severe adverse events were also documented. Worsening of pre-existing symptoms was then also investigated independently.

#### Blood safety and nutrient measures

Mean change scores from baseline to end-of-trial were calculated and log-transformed for those participants who supplied blood at baseline and end of the trial. Analysis of covariance was conducted on the logged data, controlling for baseline.

## Results

Of the 622 women who were screened, 90 (14.3%) provided informed consent and 88 (13.9%) enrolled, of which 44 were randomised to micronutrients and 44 were allocated to active placebo. Nine (20.5%) participants from the micronutrient group and eight (18.2%) participants from the active placebo group dropped out. The drop-out rate of 19.3% did not include four participants who gave birth before trial completion.

Apart from micronutrient participants being younger than active placebo participants and more micronutrient participants than active placebo participants reporting a possible alcohol dependence in the 12 months before the current pregnancy, the two groups were well matched (Table 2).

**Table 2:** Baseline demographic information and clinical characteristics for both treatment groups

	Micronutrients (*n* = 44)		Active placebo (*n* = 44)	
Characteristic	Mean or *n*	±s.d. or %	Mean or *n*	±s.d. or %
Age (years), mean (±s.d.)*	29.74	5.3	32.31	4.7
Ethnic origin, *n* (%)				
Ethnic minority (Māori, Pacific, Asian, Middle Eastern, South American, South African)	7	15.9	13	29.5
New Zealand and other European	37	84.1	31	70.5
Socioeconomic status,ᵃ mean (±s.d.)	47.20	17.3	54.05	15.8
Household income, *n* (%)				
Low: $0–40 000	11	25.0	6	13.6
Middle: $40 000–$80 000	12	27.3	20	45.5
Upper: >$80 000	21	47.7	18	40.9
In a relationship, *n* (%)	34	77.3	40	90.9
Current pregnancy information				
Duration of gestation at baseline (weeks), mean (±s.d.)	19.59	4.0	18.04	3.8
Primigravida, *n* (%)	12	27.3	9	20.5
Pre-pregnancy weight (kg), mean (±s.d.)	67.12	11.67	67.85	15.0
Supplement use at any point during current pregnancy to baseline, *n* (%)	38	86.4	40	90.9
Multidimensional Scale of Perceived Social Support, mean (±s.d.)	62.93	13.9	65.32	14.0
Current clinical characteristics (based on SCID-5 Research Version)				
Major depressive episode, *n* (%)	15	34.1	15	34.1
Any current mood disorder, *n* (%)	20	45.5	22	50.0
≥2 current mood disorders (includes ‘other specified’), *n* (%)	6	13.6	4	9.1
Any current anxiety disorder, *n* (%)	24	54.6	25	56.8
≥2 current anxiety disorders (includes ‘other specified’), *n* (%)	12	27.3	12	27.3
Current mood and anxiety disorder, *n* (%)	12	27.3	14	31.8
Past clinical characteristics (SCID-5 Research Version)				
Major depressive episode, *n* (%)	32	72.7	28	63.6
Any previous mood disorder, *n* (%)	36	81.8	30	68.2
≥2 previous mood disorders (includes ‘other specified’), *n* (%)	11	25.0	6	13.6
Any previous anxiety disorder, *n* (%)	30	68.2	26	59.1
≥2 previous anxiety disorders (includes ‘other specified’), *n* (%)	17	38.6	16	36.4
Previous mood and anxiety disorder, *n* (%)	26	59.1	22	50.0
Treatment history				
Previous use of psychiatric medications, *n* (%)	27	61.4	19	43.2
Previous psychotherapy/counselling, *n* (%)	34	77.3	29	65.9
Other measures				
EPDS at baseline, mean (±s.d.)	14.00	4.1	14.50	3.2
SAPAS ≥3, *n* (%)	23	52.3	20	45.5
Possible nicotine dependence during current pregnancy (HSI)^b^, *n* (%)	1	2.3	0	0.0
Possible alcohol dependence during current pregnancy (AUDIT-C)^b^, *n* (%)	3	6.8	0	0.0
Previous possible alcohol dependence, 12 months before current pregnancy (AUDIT-C)^b^, *n* (%)*	30	68.2	20	45.5
Expectancy of treatment effect^c^, mean (±s.d.)	3.3	0.74	3.2	0.66
Helping Alliance Questionnaire^d^, mean (±s.d.)	29.1	1.6	28.8	1.9

SCID-5, Structured Clinical Interview for DSM-5 Disorders; EPDS, Edinburgh Postnatal Depression Scale; SAPAS, Standard Assessment of Personality Abbreviated Scale; HIS, Heaviness of Smoking Index; AUDIT-C, Alcohol Use Disorders Identification Test.

a. Based on the 2013 New Zealand Socio-Economic Index (NZSEI-13).

b. Indicated by a score of ≥4.

c. Ratings ranged from 1 (not at all) to 4 (very much); data missing for 14 participants.

d. Total scores range from 5 to 30, with higher scores indicating greater alliance; data missing for 18 participants.

* Significant group difference *P* < 0.05.

### Primary outcome measures

Both groups demonstrated a statistically significant reduction in symptoms on the EPDS over the trial (*χ*^2^(2) = 153.50, *P* < 0.0001) with means for both groups ending in the normal non-clinical range; however, there was no statistically significant difference between micronutrient and active placebo (*χ*^2^(1) = 2.68, *P* = 0.1018; effect size 0.12) groups in the time and treatment only model (Table 4). Over three-quarters of participants (77.3% in the micronutrient group and 72.7% in the active placebo group) were considered to be recovered by the end of the trial (Table 3).

**Table 3:** Treatment response based on the Edinburgh Postnatal Depression Scale reliable change index from screening to the last time point available in the trial

Category of change	Micronutrients (*n* = 44)		Active placebo (*n* = 44)	
	*n*	%	*n*	%
Recovered (>4-point decrease and EPDS ≤12)	34	77.3	32	72.7
Improved but not recovered (>4-point decrease but EPDS still above ≥13)	3	6.8	4	9.1
No change (<4-point change, ±)	5	11.4	7	15.9
Deteriorated (>4-point increase)	2	4.5	1	2.3

EPDS, Edinburgh Postnatal Depression Scale.

**Fig. 2:**
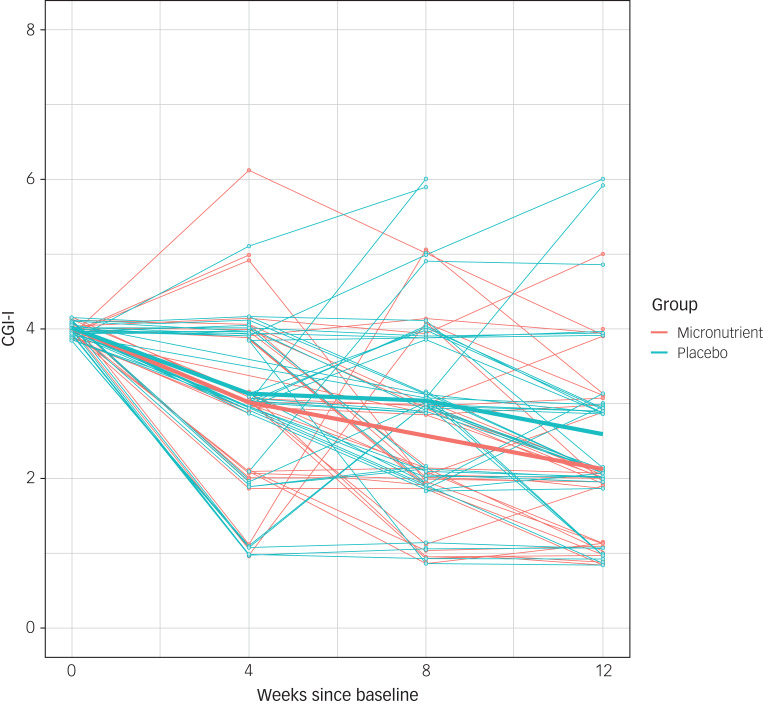
Individual and average trajectories on the Clinical Global Impression – Improvement Scale (CGI-I) for participants in the micronutrient and active placebo group over the course of the trial.

Based on the time and treatment only model, both treatment groups demonstrated a statistically significant improvement on the CGI-I over time (*χ*^2^(2) = 99.52, *P* < 0.0001). There were also statistically significantly greater improvements in the micronutrient group compared with the active placebo group (*χ*^2^(1) = 5.45, *P* = 0.0196; effect size 0.39) (Table 4 and Fig. 2).

**Table 4:** Observed baseline and post-treatment change data and Cohen's *d* on primary and secondary outcomes for participants who provided both baseline and post-treatment data, and time and treatment only model estimates for the expected difference over the 12-week study period (on the log-scale, except for binary responses)

	Micronutrients				Active placebo								
	Baseline (*n* = 43)		Raw change (*n* = 32)		Baseline (*n* = 43)		Raw change (*n* = 36)						
Outcome	Mean	±s.d.	Mean	±s.d.	Mean	±s.d.	Mean	(±s.d.)	Cohen's *d*	Effect^c^	95% CI^c^	*P* (group)^d^	*n*(obs)^e^
EPDS (self-report depression)	13.95	4.08	−7.41	5.69	14.42	3.22	−6.78	4.76	0.12	0.165	(−0.033 to 0.363)	0.1018	478
CGI-I (clinician-rated)			2.13	1.10			2.59	1.28	0.39	0.245	(0.040–0.451)	**0.0196**	222
CGI-I (binary)^a^			68.8%				51.4%			1.360	(−0.167 to 2.880)	0.1267	222
M-CGI-I (self-report)			2.25^b^	1.14			3.08^b^	1.42	0.64	0.328	(0.101–0.555)	**0.0052**	71
M-CGI-I (binary)^a^			68.8%				38.5%			1.259	(0.292–2.279)	**0.0103**	71
GAF (global functioning)	60.93	6.84	11.22	8.13	59.49	6.63	9.57	9.00	0.19	−0.049	(−0.095 to −0.003)	**0.0359**	308
MADRS (clinician-rated depression)	18.23	7.96	−8.94	7.67	18.07	7.05	−7.78	7.40	0.15	0.085	(−0.156 to 0.324)	0.4893	305
SF-12 (quality of life)	31.58	5.81	2.16	5.86	30.83	4.63	3.50	5.41	0.24	0.000	(−0.066 to 0.066)	0.9997	293
PSS (stress)	22.36	5.20	−7.68	7.57	22.59	5.02	−7.14	6.48	0.08	0.034	(−0.136 to 0.204)	0.6968	289
PSQI (sleep)	9.09	3.38	−2.22	3.40	10.44	4.26	−2.28	3.95	0.02	0.202	(0.029–0.375)	**0.0224**	294
DERS-SF (emotional regulation)	46.33	12.10	−9.03	12.67	46.98	10.78	−9.58	9.81	0.05	0.009	(−0.083 to 0.101)	0.8555	294
GAD-7 (anxiety)	9.19	4.31	−5.47	5.67	9.91	4.52	−5.14	5.13	0.06	0.204	(−0.030 to 0.439)	0.0871	477
DASS (depression, anxiety and stress)	21.07	10.04	−10.66	11.83	19.51	9.81	−8.72	7.83	0.20	0.174	(−0.082 to 0.431)	0.1824	476
Suicidal ideation^f^	0.74	0.98	−0.47	1.02	0.47	0.85	−0.16	1.07	0.29	0.038	(−0.119 to 0.196)	0.6346	305
Self-harm^g^	0.51	0.74	−0.38	0.71	0.42	0.63	−0.31	0.47	0.12	0.056	(−0.051 to 0.163)	0.3073	476

Results in bold are statistically significant (*P* < 0.05). A total of 83–85 observations were used for sample statistics at the baseline and 68–69 were used for sample statistics at the end of the 12-week study period. Effect, confidence interval and *P*-value are based on models with *n* observations. EPDS, Edinburgh Rating of Depression Scale; CGI-I, clinician rating of Clinical Global Impression – Improvement Scale; M-CGI-I, self-report or modified CGI-I; GAF, Global Assessment Functioning; MADRS, Montgomery–Åsberg Depression Rating Scale; SF-12, Short-Form Health Survey; PSS, Perceived Stress Scale; PSQI, Pittsburgh Sleep Quality Index; DERS-SF, Difficulties in Emotion Regulation Scale Short-Form; GAD-7, Generalised Anxiety Disorder-7; DASS, Depression, Anxiety and Stress Scale.

a. For binary responses (responder, non-responder), binomial generalised linear models with logit-link functions were applied, and estimated effects and confidence intervals are on the logit scale; % refers to percentage of responders at the end of the trial.

b. Average CGI-I score at the end of the trial. For the raw change, a decrease in score (−) is identified as an improvement for all measures except for the SF-12 and GAF, where an increase (+) identifies improvement in quality of life and global functioning.

c. Effects, on the log-scale, estimated from the constrained longitudinal data analysis (cLDA) model with all observations included reflecting difference between changes in the micronutrient and active placebo groups at the end of the trial. Positive difference means greater effect for the micronutrient group as compared with the active placebo group, with the exception of the GAF and SF-12.

d. Statistical significance for effect of treatment group based on cLDA model with all observations included. Based on a likelihood ratio *χ*^2^-test (d.f. = 1) comparing models with and without the treatment variable. The effect of time is statistically significant for all outcomes (*P* < 0.001).

e. Number of individual non-missing observations included in the model for 86 participants.

f. Measured using item 10 of the MADRS, with scores ranging from 0 (enjoys life) to 6 (explicit plans for suicide).

g. Measured using item 10 of the EPDS, with scores ranging from 0 (never) to 3 (yes, quite often).

The CGI-I was also used to determine responder status at the end of the trial. Based on end-of-trial data, CGI-I data were available for 69 (78.4%) participants, with 22 (68.8%) participants in the micronutrient group and 19 (51.4%) in the active placebo group considered to have responded to the intervention, although this difference was not statistically significant at the end of the study (*χ*^2^(1) = 1.4931, *P* = 0.2217; odds ratio 2.08, 95% CI 0.78–5.74).

### Auxiliary analyses on primary outcomes

After adjusting for covariates, the effect of treatment on the EPDS became statistically significant (*χ*^2^(7) = 15.96, *P* = 0.0255), with SAPAS score (*χ*^2^(3) = 8.74, *P* = 0.0329) statistically significantly modifying the effect of treatment (Table 5). Further, those with a history of psychiatric medications and those who were younger showed significantly greater change on the EPDS if taking micronutrients rather than active placebo (*t*(458.6) = −2.367, *P* = 0.0183 and *t*(457.6) = 2.072, *P* = 0.0388, respectively) (Table 6).

**Table 5:** Estimated treatment effect and statistical significance of covariates for the covariate-adjusted model

Outcome	Effect^d^	95%CI^d^	*P* (group)^e^	*P*(NZSEI)	*P* (personality difficulties)^f^	*P* (past psychiatric medications)	*P* (social support)^g^	*P* (age)	*P* (gestational age)
EPDS	0.331	(0.006–0.656)	**0.0255**	0.0995	**0.0329**	0.0768	0.3533	0.1420	0.1534
CGI-I (clinician)	0.388	(0.116–0.661)	**0.0053**	0.0748	**0.0125**	**0.01**	0.572	0.2424	0.5517
CGI-I (binary)^a^	3.180	(−0.008 to 6.360)	**0.0134**	0.8481	0.5983	0.5595	0.9874	0.9524	0.9865
M-CGI-I (self-report)	0.355	(0.104–0.605)	0.0501	0.0515	0.1176	0.0778	0.5319	0.2866	0.7541
M-CGI-I (binary)^a^	0.285	(0.031–0.538)	**0.0098**	**0.0015**	**0.0298**	**0.0278**	**0.0270**	0.2120	0.9225
GAF	−0.075	(−0.149 to 0.000)	**0.0069**	0.1704	**0.0056**	**0.0408**	0.5397	0.5840	0.0615
MADRS	0.446	(0.064–0.827)	**0.0013**	0.2904	**0.0002**	**0.0005**	0.6563	0.6249	**0.0001**
SF-12	−0.053	(−0.160 to 0.055)	0.0827	0.9615	0.3005	0.0834	0.1515	0.2275	**0.0001**
PSS	0.301	(0.020–0.583)	**0.0012**	0.7146	**0.007**	0.4515	**0.0146**	0.3618	0.0566
PSQI	0.578	(0.294–0.862)	**0.0017**	**0.0033**	0.0617	0.2872	0.4506	0.2030	**0.0001**
DERS-SF	0.156	(0.002–0.309)	0.1997	0.6653	**0.0111**	0.7695	**0.0371**	0.7358	**0.0112**
GAD-7	0.057	(−0.326 to 0.439)	**0.0283**	0.7147	**0.04**	**0.0425**	0.1649	0.2473	**0.0126**
DASS	0.234	(−0.184 to 0.652)	**0.0327**	0.4102	**0.0357**	**0.0227**	0.1545	0.1275	**0.0037**
Suicidal ideation^b^	−0.001	(−0.254 to 0.251)	0.2999	0.4359	0.6165	0.3935	**0.0156**	0.1564	0.2592
Self harm^c^	−0.055	(−0.227 to 0.118)	**0.0275**	0.1858	0.0658	0.1115	**0.0051**	0.1983	**0.0294**

The effect of time is statistically significant for all outcomes (*P* < 0.0005) except for the DERS-SF and suicidal ideation. The *P*-values show whether a covariate had a statistically significant effect on the outcome variables. Results in bold are statistically significant (*P* < 0.05). NZSEI, New Zealand Socio-Economic index; EPDS, Edinburgh Rating of Depression Scale; CGI-I, clinician rating of Clinical Global Impression – Improvement Scale; M-CGI-I, self-report or modified CGI-I; GAF, Global Assessment Functioning; MADRS, Montgomery–Åsberg Depression Rating Scale; SF-12, Short-Form Health Survey; PSS, Perceived Stress Scale; PSQI, Pittsburgh Sleep Quality Index; DERS-SF, Difficulties in Emotion Regulation Scale Short-Form; GAD-7, Generalised Anxiety Disorder-7; DASS, Depression, Anxiety and Stress Scale.

a. For binary responses (responder, non-responder), binomial generalised linear models with logit-link functions were applied, and the estimated effects and confidence intervals are on the logit scale.

b. Measured using item 10 of the MADRS, with scores ranging from 0 (enjoys life) to 6 (explicit plans for suicide).

c. Measured using item 10 of the EPDS, with scores ranging from 0 (never) to 3 (yes, quite often).

d. Marginal (i.e. averaged over the covariates) effect from the maximal mixed-effects model reflecting the difference between changes in the micronutrient group and the active placebo group at the end of the trial. Positive difference means greater effect for the micronutrient group as compared with the active placebo group for all measures, except for the GAF and SF-12.

e. Statistical significance for the effect of treatment group based on comparing covariate-adjusted constrained longitudinal data analysis (cLDA) models with and without groups via a likelihood ratio *χ*^2^-test (d.f. = 7). Same for covariate effects (d.f. = 3).

f. Measured using a score of ≥3 on the Standardised Assessment of Personality – Abbreviated Scale (SAPAS).

g. Measured with the Multidimensional Scale of Perceived Social Support (MSPSS).

**Table 6:** Estimated treatment-modifying effect and its statistical significance (in parenthesis) of covariates based on the covariate-adjusted model

Outcome	NZSEI	Personality difficulties^d^	Past psychiatric medications	Social support^e^	Age	Gestational age
EPDS	0.0014 (0.1599)	−0.0008 (0.2038)	**−0.023 (0.0183)**	−0.0427 (0.142)	**0.0003 (0.0388)**	0.0044 (0.4068)
CGI-I (clinician)	−0.0006 (0.3247)	**−0.0458 (0.0122)**	−0.0255 (0.1529)	−0.0004 (0.5444)	0.0033 (0.1297)	0.0002 (0.8855)
CGI-I (binary)^a^	25.7535 (0.057)	4.4915 (0.271)	2.4061 (0.518)	3.9523 (0.796)	−7.0798 (0.702)	6.2194 (0.712)
M-CGI-I (self-report)	−1.4154 (0.0809)	**−0.0511 (0.0425)**	−0.2491 (0.3181)	−0.7268 (0.4790)	1.9167 (0.1371)	0.9496 (0.5043)
M-CGI-I (binary)^a^	**14.3206 (0.0171)**	**3.2353 (0.0366)**	3.2794 (0.0714)	**14.7076 (0.0473)**	−10.9366 (0.1398)	−2.67 (0.7142)
GAF	0 (0.286)	**0.0001 (0.0167)**	**0.0098 (0.0288)**	0.0088 (0.7614)	0 (0.4962)	−0.0003 (0.9718)
MADRS	0.0032 (0.076)	**−0.0011 (0.0021)**	**−0.0645 (0.0061)**	−0.0564 (0.2542)	0.0006 (0.2168)	0.003 (0.0987)
SF-12	−0.0003 (0.9321)	0 (0.1339)	**0.0087 (0.0258)**	0.0128 (0.4514)	**−0.0003 (0.0496)**	−0.0014 (0.5461)
PSS	0.0029 (0.87)	**−0.0001 (0.0029)**	−0.0454 (0.1514)	**−0.0215 (0.0017)**	0.0012 (0.0872)	**0.0031 (0.048)**
PSQI	0.0043 (0.3785)	−0.0004 (0.6349)	−0.0072 (0.1433)	−0.022 (0.3043)	0.0007 (0.7691)	**−0.0005 (0.0027)**
DERS-SF	0.0015 (0.9079)	0 (0.1067)	−0.0133 (0.33)	−0.0079 (0.1135)	0.0005 (0.2802)	0.0011 (0.0519)
GAD-7	−0.0016 (0.2741)	−0.0007 (0.0695)	**−0.0386 (0.0166)**	**−0.051 (0.0247)**	0.001 (0.7768)	0.0007 (0.4183)
DASS	0.0005 (0.1797)	−0.001 (0.1347)	**−0.0346 (0.0072)**	**−0.0622 (0.0412)**	0.0008 (0.3578)	0.0025 (0.8223)
Suicidal ideation^b^	−0.0011 (0.4902)	−0.0003 (0.3262)	−0.0135 (0.3385)	−0.0130 (0.0770)	0 (0.1895)	0.0022 (0.4291)
Self harm^c^	−0.0014 (0.2061)	0.0004 (0.6052)	−0.0049 (0.1532)	**−0.0136 (0.0196)**	−0.0001 (0.5022)	0.0008 (0.101)

The treatment-modifying effects are based on the covariate×treatment×time coefficient in the model and are estimated difference in weekly changes, on the log-scale. Negative effects correspond to faster improvement for the micronutrient group as compared with the active placebo group, except for the SF-12, GAF and the binary CGI-I and M-CGI-I. The reported *P*-values show whether that covariate had a significant effect on the effect of treatment with regard to the outcome. Results in bold are statistically significant (*P* < 0.05). NZSEI, New Zealand Socio-Economic index; EPDS, Edinburgh Rating of Depression Scale; CGI-I, clinician rating of Clinical Global Impression – Improvement Scale; M-CGI-I, self-report or modified CGI-I; GAF, Global Assessment Functioning; MADRS, Montgomery–Åsberg Depression Rating Scale; SF-12, Short-Form Health Survey; PSS, Perceived Stress Scale; PSQI, Pittsburgh Sleep Quality Index; DERS-SF, Difficulties in Emotion Regulation Scale Short-Form; GAD-7, Generalised Anxiety Disorder-7; DASS, Depression, Anxiety and Stress Scale.

a. For binary responses (responder, non-responder), binomial generalised linear models with logit-link functions were applied, and the estimated effects are on the logit scale. For CGI-I (binary) and M-CGI-I (binary), the modifying effect refers to the difference at the end-point only.

b. Measured using item 10 of the MADRS, with scores ranging from 0 (enjoys life) to 6 (explicit plans for suicide).

c. Measured using item 10 of the EPDS, with scores ranging from 0 (never) to 3 (yes, quite often).

d. Measured using ≥3 on the Standardised Assessment of Personality – Abbreviated Scale (SAPAS).

e. Measured with the Multidimensional Scale of Perceived Social Support (MSPSS).

**Fig. 3:**
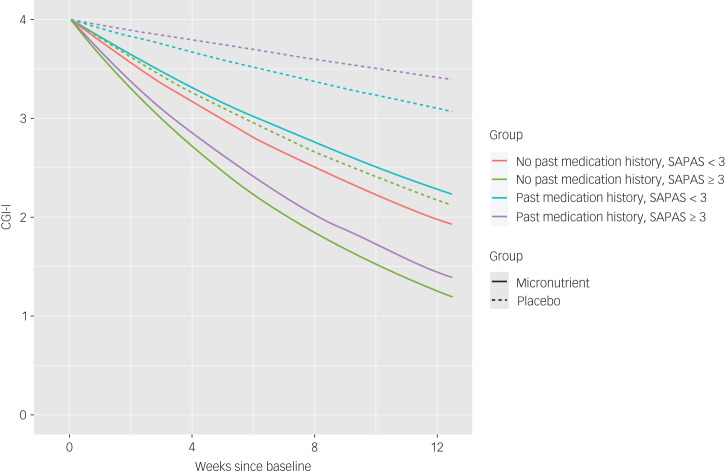
Predicted improvements based on the clinician-rated Clinical Global Impression – Improvement Scale (CGI-I) over the course of the trial, for participants in the micronutrient and active placebo group with and without personality difficulties (Standardised Assessment of Personality – Abbreviated Scale (SAPAS)) and with and without a history of psychiatric medication. The predictions have been adjusted for age of 30 years, gestational age of 30 weeks, New Zealand Socio-Economic Index of 50, Multidimensional Scale of Perceived Social Support score of 65.

The effect of treatment on the CGI-I became even more accentuated after adjusting for covariates (*χ*^2^(7) = 20.08, *P* = 0.0053), with SAPAS score (*χ*^2^(3) = 8.76, *P* = 0.0125) and past history of taking psychiatric medications (*χ*^2^(3) = 9.21, *P* = 0.0100) statistically significantly modifying the outcome (Table 5). Further, those with a personality difficulty were more likely to respond if randomised to micronutrients over active placebo (*t*(152.6) = −2.535, *P* = 0.0122) (Table 6). Figure 3 illustrates the differential effect of past medications and presence/absence of a personality difficulty on overall improvement over time on the CGI-I.

### Other treatment-related outcomes

Other outcomes showed that, based on the time and treatment only model estimates for the expected difference without adjusting for covariates, the micronutrient group had statistically significantly greater improvement over time on self-reported sleep (*P* = 0.0224) and clinician-rated GAF (*P* = 0.0359), compared with the active placebo group (see Supplementary Material available at https://doi.org/10.1192/bjo.2024.706 for graphs illustrating change on these variables over time), with no group differences on clinician-measured depression (MADRS), perceived stress, quality of life, emotional regulation, anxiety, DASS-21 score, suicidal ideation and self-harm, with both groups improving on all measures (Table 4). When adjusting for covariates, statistically significant group differences were observed on most secondary measures, with lack of personality difficulties, no history of past psychiatric medications, higher gestational age and better social support identified as the most common significant moderators, leading to the best outcomes regardless of treatment received (Table 5). However, those participants with personality difficulties, a past history of medications and better social support generally showed greater benefit if randomised to micronutrients compared with active placebo (Table 6).

When participants rated their own improvement at the end of the trial using the M-CGI-I (*n* = 71), there was also a significant group difference (*P* = 0.0052, effect size 0.636) (Table 4), with statistically significantly more participants in the micronutrient group identified as responders (22/32; 68.8%) than in the active placebo group (15/39; 38.5%) (*χ*^2^(1) = 6.581, *P* = 0.0103; odds ratio 3.52, 95% CI 1.34–9.76; number needed to treat: 3) (Table 4).

**Fig. 4:**
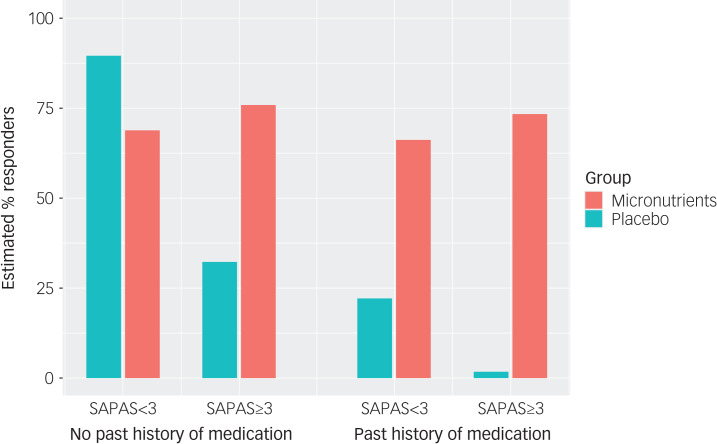
Predicted percentage of responders (much to very much improved) on the self-report modified Clinical Global Impression – Improvement Scale (M-CGI-I) for 71 participants who completed the M-CGI-I with and without personality difficulties (Standardised Assessment of Personality – Abbreviated Scale (SAPAS)) and with and without a history of taking psychiatric medication. The predictions have been adjusted for age of 30 years, gestational age of 30 weeks, New Zealand Socio-Economic Index of 50, Multidimensional Scale of Perceived Social Support score of 65.

To contextualise the outcomes from a clinical perspective (i.e. responder status based on binary patient characteristics), we present the model prediction for those groups, adjusted for other covariates, in Fig. 4. This modelling shows that for those identified with both personality difficulties and a past history of medications, exposure to micronutrients are necessary to ensure a treatment response.

#### Adverse effects

Overall, there was no statistically greater emergence of adverse effects reported by participants in the micronutrient group compared with the active placebo group (Table 7). We also considered whether there was any worsening of symptoms that were present before the intervention and, other than the micronutrient group reporting a temporary greater change in weight gain (not supported by our anthropometric data), there were no group differences in worsening of symptoms present before study entry as a consequence of the intervention (Table 8). Further, in those cases where prior symptoms did get worse, the worsening was typically transient.

**Table 7:** Treatment emergent adverse events reported on the adapted Antidepressant Side Effect Checklist by at least 5% of participants per treatment group during the trial phase

Adverse event	Micronutrients		Active placebo				
	Adverse event not present at baseline	Emergence of adverse event	Adverse event not present at baseline	Emergence of adverse event			
	*n* (%)	*n* (%)	*n* (%)	*n* (%)	Odds ratio	95% CI	*P*-value^c^
Dry mouth	24 (54.6)	13 (54.2)	26 (59.1)	13 (50.0)	1.18	(0.34–4.14)	0.7851
Drowsiness	16 (36.4)	9 (56.3)	12 (27.3)	4 (33.3)	2.48	(0.43–16.48)	0.2761
Insomnia	14 (31.8)	9 (64.3)	11 (25.0)	7 (63.6)	1.03	(0.14–7.04)	>0.9999
Blurred vision	32 (72.7)	6 (18.8)	34 (77.3)	4 (11.8)	1.72	(0.36–9.22)	0.5052
Headache	14 (31.8)	6 (42.9)	12 (27.3)	7 (58.3)	0.55	(0.09–3.26)	0.6951
Constipation	18 (40.9)	11 (61.1)	18 (40.9)	11 (61.1)	1.00	(0.21–4.67)	>0.9999
Diarrhoea	39 (88.6)	18 (46.2)	33 (75.0)	8 (24.2)	2.64	(0.88–8.55)	0.0841
Increased appetite	17 (38.6)	9 (52.9)	18 (40.9)	9 (50.0)	1.12	(0.25–5.16)	>0.9999
Decreased appetite	30 (68.2)	7 (23.3)	31 (70.5)	5 (16.1)	1.57	(0.37–7.21)	0.5339
Nausea or vomiting/stomach pain^a^	26 (59.1)	19 (73.1)	20 (45.5)	9 (45.0)	3.23	(0.82–13.70)	0.0716
Problems with urination	35 (79.6)	5 (14.3)	38 (86.4)	5 (13.2)	1.10	(0.23–5.29)	>0.9999
Problems with sexual function	33 (75.0)	5 (15.2)	34 (77.3)	4 (11.8)	1.33	(0.26–7.44)	0.7337
Palpitations	29 (65.9)	7 (24.1)	28 (63.6)	5 (17.9)	1.45	(0.34–6.75)	0.7470
Feeling light-headed on standing	15 (34.1)	5 (33.3)	17 (38.6)	10 (58.8)	0.36	(0.06–1.82)	0.1777
Feeling like the room is spinning/dizziness^a^	32 (72.7)	9 (28.1)	30 (68.2)	8 (26.7)	1.07	(0.30–3.85)	>0.9999
Sweating	24 (54.6)	9 (37.5)	29 (65.9)	8 (27.6)	1.56	(0.42–5.90)	0.5575
Increased body temperature	26 (59.1)	19 (73.1)	23 (52.3)	13 (56.5)	2.06	(0.54–8.25)	0.2475
Tremor	40 (90.9)	1 (2.5)	40 (90.9)	5 (12.5)	0.18	(0.00–1.75)	0.2007
Disorientation	36 (81.8)	5 (13.9)	36 (81.8)	7 (19.4)	0.67	(0.15–2.78)	0.7531
Yawning	18 (40.9)	8 (44.4)	18 (40.9)	8 (44.4)	1.00	(0.22–4.51)	>0.9999
Weight gain	22 (50.0)	16 (72.7)	18 (40.9)	12 (66.7)	1.32	(0.28–6.40)	0.7385
Itching^b^	26 (59.1)	12 (46.2)	27 (61.4)	12 (44.4)	1.07	(0.32–3.61)	>0.9999
Rash^b^	36 (81.8)	7 (19.4)	38 (86.4)	8 (21.1)	0.91	(0.24–3.28)	>0.9999
Numbness, pain, burning or tingling in hands, feet or legs^b^	33 (75.0)	12 (36.4)	27 (61.4)	10 (37.0)	0.97	(0.30–3.20)	>0.9999
Other adverse events reported^a^	212	15	215	22			
Restless legs	43 (97.9)	0 (0.0)	43 (97.9)	4 (9.3)	0.00	(0.00–1.47)	0.1162
Breathlessness	44 (100.0)	1 (2.3)	43 (97.9)	4 (9.3)	0.23	(0.00–2.46)	0.2024
Indigestion	43 (97.9)	9 (20.5)	42 (95.5)	6 (14.3)	1.58	(0.45–6.01)	0.5710
Thrush	42 (95.5)	2 (4.8)	44 (100.0)	2 (4.6)	1.05	(0.07–15.12)	>0.9999
Any lower body pain (including sciatica, symphysis pubic dysfunction)	40 (90.9)	3 (7.5)	43 (97.7)	6 (14.0)	0.50	(0.08–2.57)	0.4853

ASEC, Antidepressant Side Effect Checklist.

a. Adverse events reported in addition to those listed in the ASEC, not standardly measured at baseline.

b. Adverse events added to the ASEC.

c. Comparisons between number of participants who experienced emergence of an adverse event during the trial and participants who did not report an emergence in each treatment group, using the Fisher exact test. Note that those who reported any adverse event at baseline were not included in this comparison.

**Table 8:** Total number of participants in each group reporting a worsening of symptoms present before commencement of the trial, based on the adapted Antidepressant Side Effect Checklist

Adverse event	Micronutrients				Active placebo				*P-*value
	Adverse event present at baseline		Worsened		Adverse event present at baseline		Worsened		
	*n*	%	*n*	%	*n*	%	*n*	%	
Blurred vision	12	27.7	3	25.0	10	22.7	2	20.0	0.781
Constipation	26	59.1	8	30.8	26	59.1	12	46.2	0.254
Decreased appetite	14	31.8	5	35.7	13	29.5	2	15.4	0.228
Diarrhoea	5	11.4	2	40.0	11	25.0	5	45.5	0.838
Disorientation	8	18.2	2	25.0	8	18.2	1	12.5	0.521
Drowsiness	28	63.6	9	32.1	32	72.7	7	21.9	0.370
Dry mouth	20	45.5	8	40.0	18	40.9	4	22.2	0.239
Feeling light headed on standing	29	65.9	5	17.2	27	31.4	6	22.2	0.639
Feeling like the room is spinning	12	27.3	5	41.7	14	31.8	3	21.4	0.265
Headache	30	38.2	7	23.3	32	72.7	6	18.8	0.658
Increased appetite	27	31.4	6	22.2	26	59.1	5	19.2	0.788
Increased body temperature	18	40.9	5	27.8	21	47.7	2	9.5	0.139
Insomnia	30	38.2	10	33.3	33	75.0	15	45.5	0.326
Itching	18	40.9	4	22.2	17	38.6	2	11.8	0.412
Nausea	18	40.9	5	27.8	24	54.5	4	16.7	0.385
Numbness/tingling	11	25.0	1	9.1	17	38.6	7	41.2	0.066
Palpitations	15	34.1	4	26.7	16	36.4	4	25.0	0.916
Problems with sexual functioning	11	25.0	2	18.2	10	22.7	1	10.0	0.684
Problems with urination	9	20.5	3	33.3	6	13.6	0	0.0	0.229
Rash	8	18.2	1	12.5	6	13.6	1	16.7	0.593
Sweating	20	45.5	6	30.0	15	34.1	3	20.0	0.503
Weight gain	22	50.0	10	45.5	26	59.1	4	15.4	**0.022**
Yawning	26	59.1	3	11.5	26	59.1	2	7.7	0.638

Participants who endorsed any symptom on the adapted ASEC (score of ≥1) at baseline that worsened at any point throughout the trial were included in the analysis, to determine whether there were any differences between the groups in worsening of pre-existing symptoms. ASEC, Antidepressant Side Effect Checklist.

An adverse event was considered serious when the participant presented to emergency services, attempted suicide or death occurred. Of all adverse events reported, three (1.1%) serious adverse events (SAEs) occurred in the micronutrient group (migraine, rash and spotting), each resulting in presentation to hospital. Only the rash was deemed potentially related to the intervention. Of all adverse events reported in the active placebo group, ten (4.2%) were considered serious and resulted in hospital presentation (suicide attempt, death of a neonate, mild stroke, numbness, allergic reaction, chest pain, flu-like symptoms, muscle strain and a sprained ankle). None of these events were deemed likely to be related to the intervention. Although there were fewer SAEs in the micronutrient group, there was no significant group difference (*χ*^2^(1) = 3.47, *P* = 0.062; odds ratio 0.285, 95% CI 0.07–1.13).

Of the 21 (47.7%) participants in the micronutrient group who did not report thoughts of suicide and/or self-harm at baseline, two (9.5%) reported their emergence at some point during the trial. Of the 24 (54.6%) active placebo participants who did not report thoughts of suicide and/or self-harm at baseline, five (20.8%) reported emergence during the trial, with no statistically significant group differences (*χ*^2^(1) = 1.0906, *P* = 0.296). There was also no statistically significant difference in the persistence of suicidal ideation between the treatment groups in participants (*n* = 43) who reported thoughts of suicidality/self-harm at baseline (*χ*^2^(1) = 1.8647, *P* = 0.172).

#### Blood safety and nutrient measures

Overall, 47 (53.4%) participants provided blood samples at both baseline and at the end of the trial: 24 (54.6%) participants in the micronutrient group and 23 (52.7%) in the active placebo group. The micronutrient group showed statistically significantly greater reduction in homocysteine compared with the active placebo group (F(1,46) = 13.66, *P* < 0.001), and statistically significantly greater increase in vitamin B12 (F(1,46) = 39.80, *P* < 0.001) and vitamin D (F(1,46) = 27.20, *P* < 0.001), all yielding large effect sizes. There were no other statistically significant group differences (Table 9).

**Table 9:** Baseline and post-treatment change data on haematology blood count and nutrient levels for participants who provided both baseline and post-treatment blood samples

Biomarker	Reference range	Micronutrients (*n* = 24)						Active placebo (*n* = 23)						ANCOVA *P*-value^a^	Effect size (Cohen's *d*)
		Baseline			Raw change			Baseline			Raw change				
		Mean	±s.d.	Median	Mean	±s.d.	Median	Mean	±s.d.	Median	Mean	±s.d.	Median		
Haematology blood count															
Haemoglobin (g/L)	115–155	121.13	10.02	119	−2.67	9.42	−2.50	121.61	9.25	123	−6.35	9.86	−7.00	0.181	0.40
Haematocrit (ratio)	0.35–0.46	0.36	0.03	0.350	−0.01	0.03	−0.01	0.36	0.03	0.360	−0.01	0.03	−0.02	0.167	0.41
MCV (fl)	80–99	90.67	3.40	91.0	0.29	2.17	1.00	90.35	3.34	89.0	0.91	1.78	1.00	0.343	−0.28
MCH (pg)	27–33	30.38	1.50	30.5	−0.08	1.25	0.00	30.57	1.50	30.0	0.04	1.11	0.00	0.679	−0.12
Platelets (×10^9^/L)	150–400	255.71	78.20	246	−10.13	42.06	−5.50	249.09	31.81	254	−13.04	29.40	−12.0	0.431	0.23
WBC (×10^9^/L)	4.0–11.0	8.57	1.65	8.55	1.22	1.52	0.95	8.17	1.93	8.20	1.42	1.61	1.70	0.637	−0.14
Neutrophils (×10^9^/L)	1.9–7.5	6.07	1.35	6.10	0.96	1.39	0.80	5.84	1.64	5.80	1.10	1.44	1.00	0.796	−0.08
Lymphocytes (×10^9^/L)	1.0–4.0	1.76	0.36	1.70	0.11	0.29	0.15	1.71	0.40	1.70	0.11	0.30	0.00	0.767	−0.09
Monocytes (×10^9^/L)	0.2–1.0	0.49	0.12	0.49	0.16	0.31	0.10	0.43	0.11	0.45	0.16	0.16	0.20	0.805	0.07
Eosinophils (×10^9^/L)	0.0–0.5	0.18	0.11	0.16	0.03	0.13	0.01	0.12^b^	0.10	0.09	0.01	0.03	0.00	0.557	0.19
Meta/myelocytes (×10^9^/L)	0.0–0.06	0.04^c^	0.03	0.03	0.03	0.04	0.01	0.04	0.03	0.03	0.04	0.05	0.04	0.124	−0.52
Basophils (×10^9^/L)	0.0–0.2	0.03^c^	0.02	0.03	0.00	0.03	0.00	0.02^b^	0.02	0.02	0.01	0.03	0.01	0.883	−0.05
Other blood results															
Ceruloplasmin (g/L)	0.15–0.60	0.44	0.08	0.42	0.01	0.07	0.01	0.44	0.08	0.41	0.03	0.06	0.02	0.444	−0.23
Homocysteine, plasma (*μ*mol/L)	5.0–15.0	4.43^d^	1.13	4.20	−0.44	0.86	−0.50	4.32^d^	1.15	4.20	0.55	0.96	0.60	**<0.001**	−1.16
Nutrient levels															
Iron (*μ*mol/L)	10–30	17.87^c^	5.54	19.0	−4.09	7.18	−3.00	19.95^b^	5.10	20.5	−1.77	12.75	−3.50	0.182	−0.41
Ferritin (*μ*g/L)	20–200	39.96^c^	42.33	24.0	−24.39	32.82	−14.0	51.27^b^	58.71	36.0	−33.14	46.89	−22.5	0.591	−0.164
Vitamin B12 (pmol/L)	130–650	269.92	123.41	231	96.67	93.03	110	263.91	87.78	249	−45.70	69.32	−32.0	**<0.001**	1.99
Plasma 25-hydroxy vitamin D (nmol/L)	50–150	78.42	31.13	67.5	50.38	32.77	43.0	74.48	22.99	73.0	5.91	36.43	9.00	**<0.001**	1.45
Plasma copper, total (*μ*mol/L)	11.0–22.0	30.60^c^^,^^d^	5.60	29.6	1.43	4.00	1.80	29.83^d^	5.35	29.4	1.80	3.96	0.90	0.971	0.01
Plasma zinc, total (*μ*mol/L)	10.0–17.0	9.78^c^^,^^d^	1.36	9.40	−0.38	1.66	−0.60	9.77^d^	0.98	9.90	−0.91	119	−0.70	0.282	0.32
Other biometric measures															
Weight (kg)		81.9	20.0	76.8	2.65	1.18	2.55	78.8	15.6	75.9	2.2	1.3	2.30	0.329	0.36
Blood pressure: upper		117	10.4	119	0.80	7.58	2.50	117	17.8	115	−2.25	7.6	2.50	0.464	0.40
Blood pressure: lower		69.3	9.9	68.5	2.75	7.03	1.00	70.0	10.6	68.0	1.38	12.0	1.50	0.687	0.14
Pulse		85.3	10.9	83.5	−0.25	11.5	3.50	85.5	11.3	81.5	−1.00	8.4	−2.00	0.554	0.07

Results in bold are statistically significant (*P* < 0.05). ANCOVA, analysis of covariance; MCV, mean corpuscular volume; MCH, mean corpuscular haemoglobin; WBC, white blood cell count.

a. Based on logarithmic transformation scale (log10).

b. *n* = 22.

c. *n* = 23.

d. Outside reference range.

#### Adherence and blinding

Adherence with treatment was excellent (92%). On average, the micronutrient group reported taking 92.8% (±7.65 s.d.) of the treatment dose, and the active placebo group reported taking 92.1% (±10.03 s.d.) of the treatment dose. The self-reported number of capsules taken in both groups was comparable to the number found from the count of remaining capsules that were returned (90.2%). Blinding to treatment condition was maintained. Clinicians correctly identified treatment allocation for 53.4% of participants, and 62% of participants correctly identified their treatment allocation.

## Discussion

This 12-week study of 88 women with moderate depressive symptoms during pregnancy aligns with previous research identifying both significant and clinical benefit from participation in research, regardless of group allocation, with specific global improvement in functioning observed with the micronutrients over active placebo.^52–54^ Both groups improved statistically significantly on patient-measured depression, with no group difference based on the EPDS (*P* = 0.1018; effect size 0.12), with 77.3% in the micronutrient group and 72.7% in the active placebo group considered to have recovered. However, the micronutrient group demonstrated statistically significantly greater improvements in symptoms over time compared with the active placebo group, based on clinician CGI-I (*P* = 0.0196; effect size 0.39). There was also statistically greater change for those assigned to micronutrients compared with placebo on sleep and global assessment of functioning, replicating other studies of micronutrient benefit on these measures,^12,55^ and 68.8% of micronutrient participants self-rated themselves as much to very much improved (responder) versus 38.5% from the active placebo group (odds ratio 3.52; *P* = 0.011; number needed to treat: 3). There were no group differences on improvement in quality of life, clinician-measured depression, stress, self-harm, suicidal ideation and anxiety, with both groups improving on all measures. Retention in the study was good (81%), adherence was excellent (>90%), the blind was well maintained and there were no group differences in safety, emergence of suicidal ideation or side-effects. Outcomes were comparable to those obtained using psychotherapy, but achieved with much less contact, although comparison is made more difficult because the comparators for psychotherapy trials tend to be treatment as usual rather than a blinded placebo group.^56^ Also, psychotherapy studies typically have smaller sample sizes, a higher dropout rate and greater chance of bias owing to their lack of blinding. There are no medication RCTs during pregnancy to compare our results against.

Treatment outcome was moderated by personality difficulties, presence/absence of past psychiatric medications, social support and gestational age. Personality difficulties is an optional coding in the ICD-11, and is used to inform treatment and preventative care, as it can influence health status and interactions with health services.^57^ When personality difficulties were present, the effect of micronutrients was greater than the placebo effect; a differential effect observed with cognitive–behavioural therapy in patients with health anxiety,^58^ where those with personality difficulties had greater improvement in symptoms over 2 years compared with those without personality pathology. Those with personality difficulties typically face greater impairments and poorer quality of life, making them more susceptible to mental health issues.^59^ In contrast, individuals with fewer personality difficulties tend to have better overall functioning, potentially benefiting from spontaneous improvement or general clinical care. Considering the significant impact of micronutrients on neurotransmitter function and observed improvements in emotional dysregulation and aggression with micronutrient interventions,^54^ it is plausible that additional micronutrient exposure directly benefited emotional dysregulation in those with personality difficulties.

Clinical improvement was also moderated by past history of psychiatric medication use, with overall response being significantly lower in participants who had trialled psychiatric medication in the past and assigned to placebo, replicating other research using these same micronutrients for depression.^13^ Those with a history of psychiatric medication represent those who are potentially ‘treatment resistant’ and, therefore, identifying the moderating effect of this patient characteristic could lead to more targeted interventions. Also, those with greater social support were, on average, more likely to benefit from the micronutrients, a finding that has been observed with other treatment interventions.^60^ The effect of gestational age was present across both treatment groups, in that the further the mother was along in her pregnancy, the better her psychological functioning, consistent with other documented improvements in antenatal depression as a pregnancy progresses.^61^

Of note is the discrepancy between the primary outcomes. Clinician-rated scales gather information not captured sufficiently in self-report,^62^ as clinician ratings are based on multiple sources of information, including other clinician-rated and self-report questionnaires, participant comments, experiences of significant side-effects and observations of the participant at the assessment meeting. Although the CGI-I considers both positive and negative changes in symptoms, the EPDS is subjective and unidimensional, and solely focuses on negative symptoms of depression and anxiety, thereby increasing the risk of a floor effect.^63^ Therefore, the CGI-I probably allows for a more stable, accurate and global assessment of symptom change, in contrast with the brief EPDS. The more robust change observed with the CGI-I is also consistent with other micronutrient studies,^52,53^ showing that the impact of micronutrients is likely not specific to one dimension of pathology, but may better serve as an overall metabolic tune-up.^11^

Why was there a difference between the self-report ratings of the EPDS and the participant rating of improvement (M-CGI-I)? Repeated measurement in a treatment trial is known to result in regression to the mean,^64^ and this was observed with the EPDS. In contrast, the M-CGI-I was administered once at the end of the trial, and invited participants to consider their overall functioning, capturing both absence of symptoms and improvements, and therefore may have been less susceptible to this phenomenon. The fact that the results for the clinician-rated and participant-rated global impression of change (completed independently) were in general accordance is consistent with other research showing agreement between these two ratings.^31^ However, although the CGI-I measure showed a benefit, it was not as pronounced as the M-CGI-I, perhaps because the M-CGI-I is reliant on memory of baseline functioning, thereby compromising the validity of the rating, whereas the CGI-I rating was derived from consideration of all data collected across time.

Overall, symptoms of the active placebo group generally improved over the trial based on both the CGI-I and the EPDS, suggesting that regardless of treatment arm, a therapeutic benefit was experienced. Possible explanations include the general therapeutic benefit of participating in clinical trials, positive therapeutic alliance, expectation of a treatment benefit, potential benefits of iodine and riboflavin on mood, increased fluid intake from taking capsules, eating regular meals and regression to the mean. Given there was no significant difference between groups on variables that may be associated with the placebo effect (e.g. expectancy of benefit, therapeutic alliance), the larger improvements observed in the micronutrient group most likely constituted a genuine effect. The likelihood of the observed effect being a true effect is also demonstrated when looking at the moderators of treatment response (personality difficulties and past history of psychiatric medications), given these specific effects have been observed in other clinical trials.

The current findings provide preliminary evidence for the safety of broad-spectrum micronutrients during pregnancy and are consistent with other research,^11,52^ although post-surveillance reports are required. It was reassuring that suicidal ideation and self-harm did not emerge as a treatment-related side-effect. Observational follow-up of the infants born to these mothers who took micronutrients antenatally showed positive effects on infant regulation, on par with or better than typical pregnancies and superior to antidepressants,^65^ and no observed negative effects on temperament up to 1 year.^66^ Blood tests suggested that increased micronutrient intake resulted in meaningful increases in vitamins B12 and D, with other research on this sample documenting that there were fewer women developing vitamin C deficiency for those in the micronutrient group compared with placebo.^67^ The reduction in homocysteine in the micronutrient group provides insight into one potential mechanism of action in that the micronutrients may positively affect the methylation cycle, a cycle that supports the production of glutathione, an important antioxidant. Although other inflammatory markers, such as interleukins 6 and 10, were measured for a subgroup of participants, the lack of overall elevations at baseline precluded assessing whether the micronutrients had a positive impact on those inflammatory levels.^67^ Measurements of blood pressure and pulse showed no changes over time, providing further safety data for micronutrient treatment during pregnancy.

Strengths of this study included using the gold standard, fully blinded, randomised placebo-controlled trial. Treatment allocation and concealment were conducted by individuals independent of the research team to eliminate the risk of selection bias, ensuring blinding of participants, clinicians and the statistician. Prospective trial registration and publication of the study protocol increased transparency. Good adherence and retention rates suggest the micronutrients were tolerated and acceptable for these pregnant women. Finally, the prevalence of personality difficulties in the current study (48.9%) was similar to the rate typically found in the community,^68^ suggesting that the current sample was representative of the general population.

Study limitations included difficulties with recruitment and the large placebo effect, a common problem in trials testing psychological interventions in pregnancy;^69^ time to participate and concerns about the unknown safety of the micronutrients presented as significant barriers to recruitment, as well as the onset of the COVID-19 pandemic. The number of recruited participants was lower than that projected, which lowered the estimated power of the trial. However, the power analysis was based on the simplified *t*-test framework rather than on the more powerful mixed-effects linear model. Further, estimates were determined based on non-pregnant populations, given the novelty of this research. The analysis was still able to detect a statistically significant effect of treatment on the CGI-I, although it was insufficient to do so for the EPDS. One could argue that the similar change in the EPDS across both groups, with over three-quarters of participants considered to have a reliable and clinically significant change, means that although a larger sample might detect a group difference on the EPDS, it may not be that clinically meaningful. However, given this study is one of the larger clinical trials to date on symptoms of antenatal depression, and larger than some of those that inform the National Institute for Health and Care Excellence guidelines (https://www.nice.org.uk/guidance/cg192/evidence/full-guideline-pdf-4840896925), we attained a sample size that many research groups have been unable to achieve.

The majority of participants reported moderate symptoms of depression and believed that the treatment would be beneficial. The generalisability of the study findings to women with more severe symptoms/meeting diagnostic criteria for depression, or who are less optimistic regarding a potential treatment response, is limited. Generalisability is also influenced by a high percentage of the sample being of New Zealand European descent (77%), none of the women were taking psychiatric medications, the relatively high socioeconomic status of participants and small geographical representation of the sample. In retrospect, a better measure of depression could have been chosen, given that only 48% met full criteria for major depression based on the SCID-5 Clinician Version, despite receiving an EPDS score ≥13; however, comparison with other studies is important, given that the EPDS is the most widely used measure of antenatal depression.

This novel RCT is the first to study efficacy and safety of micronutrients as a treatment of symptoms of depression during pregnancy, with initial signals of some potential global benefit over and above the substantial placebo effect, with no identified risks, and possibly specific subgroups to target. However, replication with larger sample sizes, more severe pathology and more diverse population is imperative because of the preliminary nature of this study. The high placebo effect achieved with fewer side-effects than antidepressants and with positive infant outcomes^65,66^ should be welcomed by patients and clinicians trying to navigate options during pregnancy, given the lack of controlled trials of antidepressants during the antenatal period. These findings align with the growing body of literature highlighting the potential advantage of an abundant nutritional environment in improving brain health.^70^

## Supporting information

Bradley et al. supplementary material Bradley et al. supplementary material

## Data Availability

Individual participant data that underlie the results reported in this article, after de-identification, and the statistical analysis plan and analytical code will be made available following publication, on reasonable request, to researchers who provide a methodologically sound proposal. Proposals should be directed to the corresponding author, J.J.R.; to gain access, data requestors will need to sign a data access agreement.
