# Supplementary material for: Efficacy and safety of a mineral and vitamin treatment on symptoms of antenatal depression: 12-week fully blinded randomised placebo-controlled trial (NUTRIMUM)
Source: BJPsych Open. 2024 Jun 3;10(4):e119. doi: 10.1192/bjo.2024.706 (PMC11363078; doi:10.1192/bjo.2024.706)
Supplement: Bradley et al. supplementary material [file S2056472424007063sup001.docx]

Supplementary Material.

Code for Analysis in R.

In this section, we provide the R code for analysing one outcome, EPDS. Here, d2 is a *long* data-frame with one row for each person-time observation.

## loading the necessary libraries:
library(lme4)
library(lmerTest)

library(emmeans)

###

### The model without covariates:

d.in <- d2[!is.na(d2$EPDS.total),]
 d.in$GroupNum <- 2-as.numeric(d.in$Group)

 m.max <- lmer(log(EPDS.total+1) ~ TimeNum + GroupNum:TimeNum + (1|R.), data=d.in, REML=F)
 m.max.noG <- lmer(log(EPDS.total+1) ~ TimeNum + (1|R.), data=d.in, REML=F)
 m.max.noT <- lmer(log(EPDS.total+1) ~ 1 + (1|R.), data=d.in, REML=F)

 # effect of time:
 anova(m.max,m.max.noT)

# effect of the treatment group:
 anova(m.max,m.max.noG)

# the estimated effect over 12 weeks (on a log scale)
 print(round((fixef(m.max)[2]*12),4))

# the 95% CI:
 print(round(confint(m.max)[4,]*12,4))

### The model with covariates:

d2 <- d.analyse

 d.in <- d2[!is.na(d2$EPDS.total),]
 d.in$GroupNum <- 2-as.numeric(d.in$Group)

 m.max <- lmer(log(EPDS.total+1) ~ (TimeNum + GroupNum:TimeNum)*
 (Age.imp+Gestation.imp+NZ.SEI.13.score+SAPAS3plus+Past.psyc.meds+Social.support)+ (1|R.),
 data=d.in, REML=F)
 m.max.noG <- lmer(log(EPDS.total+1) ~ TimeNum*
 (Age.imp+Gestation.imp+NZ.SEI.13.score+SAPAS3plus+Past.psyc.meds+Social.support)+ (1|R.),
 data=d.in, REML=F)
 m.max.noT <- lmer(log(EPDS.total+1) ~
 Age.imp+Gestation.imp+NZ.SEI.13.score+SAPAS3plus+Past.psyc.meds+Social.support+ (1|R.),
 data=d.in, REML=F)

# The effect of treatment group:
anova(m.max,m.max.noG)

# The effect of time:
anova(m.max,m.max.noT)

Obtaining marginal effect estimator between T0 and T12 using contrasts, based on the maximal model:

$$\Delta= \left( E\left( Y | Grp=1,T=0 \right)-E\left( Y | Grp=1,T=12 \right) \right)$$

$$-(E(Y|Grp=0,T=0)-E(Y|Grp=0,T=12))$$

# The effects on a log scale:

em <- emmeans(m.max, ~ GroupNum,at=list(TimeNum=12))
 cc <- contrast(em,list(c(-1,1)))

 # the mean estimated effect:
 print(round((summary(cc))$est,4))

# the lower 95% CI limit:
 print(round((summary(cc))$est-1.96*summary(cc)$SE,4))

# the upper 95% CI limit:
 print(round((summary(cc))$est+1.96*summary(cc)$SE,4))

Supplementary Graphs.


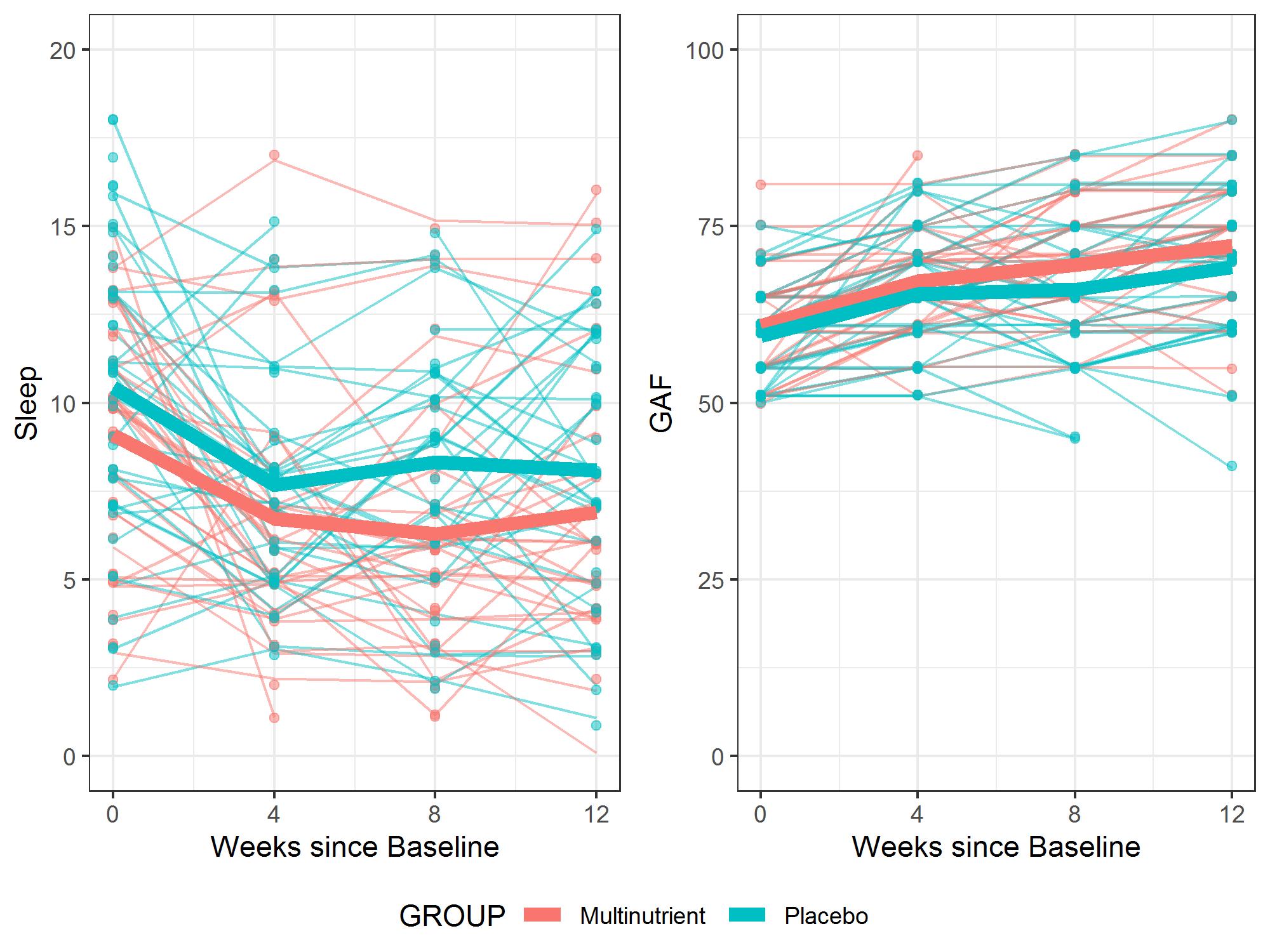


Fig. S1. Individual (thin lines) and group average (thick lines) sleep (left panel) and GAF (right panel) responses for the placebo (blue) and micronutrient (red) treatment groups respectively. The Cohen’s d in Table 4 is based on observed statistics from the pre (week 0) and post (week 12) time points only whereas the reported cLDA model (and the associated p-values) takes into account the intermediate time points (weeks 4 and 8) as well as the repeated measures nature of the experiment (some individuals have been observed more than others and these observations are weighted accordingly).
